# Discovery of a receptor guanylate cyclase expressed in the sperm flagella of stony corals

**DOI:** 10.1038/s41598-019-51224-7

**Published:** 2019-10-10

**Authors:** Yan Zhang, Yi-Ling Chiu, Chieh-Jhen Chen, Yu-Ying Ho, Chuya Shinzato, Shinya Shikina, Ching-Fong Chang

**Affiliations:** 1 Center of Excellence for the Oceans, National Taiwan Ocean University, Keelung, 20224 Taiwan; 2 Doctoral Program in Marine Biotechnology, National Taiwan Ocean University, Keelung, 20224 Taiwan; 3 Doctoral Program in Marine Biotechnology, Academia Sinica, Taipei, 11529 Taiwan; 4 Department of Aquaculture, National Taiwan Ocean University, Keelung, 20224 Taiwan; 5 Atmosphere and Ocean Research Institute, The University of Tokyo, Chiba, 277-8564 Japan; 6 Institute of Marine Environment and Ecology, National Taiwan Ocean University, Keelung, 20224 Taiwan

**Keywords:** Molecular evolution, Molecular biology, Reproductive biology

## Abstract

The receptor guanylate cyclases (rGCs) in animals serve as sensitive chemoreceptors to detect both chemical and environmental cues. In reproduction, rGCs were shown to be expressed on sperm and serve as receptors for egg-derived sperm-activating and sperm-attracting factors in some echinoderms and mammals. However, sperm-associated rGCs have only been identified in some deuterostomes thus far, and it remains unclear how widely rGCs are utilized in metazoan reproduction. To address this issue, this study investigated the existence and expression of rGCs, particularly asking if rGCs are involved in the reproduction of a basal metazoan, phylum Cnidaria, using the stony coral *Euphyllia ancora*. Six paralogous rGCs were identified from a transcriptome database of *E. ancora*, and one of the rGCs, GC-A, was shown to be specifically expressed in the testis. Immunohistochemical analyses demonstrated that *E. ancora* GC-A protein was expressed in the spermatocytes and spermatids and eventually congregated on the sperm flagella during spermatogenesis. These findings suggest that GC-A may be involved in the regulation of sperm activity and/or functions (e.g., fertilization) in corals. This study is the first to perform molecular characterization of rGCs in cnidarians and provides evidence for the possible involvement of rGCs in the reproduction of basal metazoans.

## Introduction

The receptor guanylate cyclases (rGCs), also referred to as membrane rGCs^1^, serve as sensitive chemoreceptors for detecting not only the presence of semiochemicals (e.g., peptides) but also the changes in environmental cues (e.g., temperature, pH and carbon dioxide)^1–3^. Activation of rGCs by their ligands or extracellular cues (e.g., ion) induces the production of second messenger cyclic guanosine 3′,5′-monophosphate (cGMP) which, in turn, exerts a variety of physiological functions in the ossification process and cardiovascular homeostasis, intestinal fluid-ion homeostasis/intestinal cell proliferation^4–6^, as well as sensory processing systems (e.g., vision, gustation, olfaction, and thermosensation)^1,3,7,8^.

In reproduction, rGCs play important roles in fertilization processes, particularly in guiding sperm to the egg in some animals. For instance, in echinoderms (e.g., sea urchin and starfish), rGCs expressed on the sperm flagella act as chemoreceptors for egg-derived sperm-activating and attracting factors (SAAF) and adjust the mobility and migration of sperm to fertilize the eggs^8–11^. Studies in mammals have also shown that the rGCs expressed on sperm are involved in the regulation of sperm attraction and acrosome reaction^12–17^. In addition to sperm guidance, specific types of mammalian rGCs (e.g., GC-A or GC-B) and their ligands (e.g., atrial natriuretic peptide or C-type natriuretic peptide) also regulate several reproductive functions, including testicular steroidogenesis^18^, blood-testis barrier dynamics^19^, and oocyte meiosis^20–24^. These studies thus illustrate the significance of rGCs in the reproductive processes of both sexes.

To date, many forms of rGCs have also been identified and characterized in a variety of animals, including fish^25–27^, and some model invertebrates, such as *C. elegans* and *Drosophila*^7,28^; however, to the best of our knowledge, the involvement of rGCs in reproduction has only been demonstrated in some deuterostomes (echinoderms and mammals)^10^. Reproduction-associated rGCs, which are expressed in germline cells and/or gonadal somatic cells and play important roles in reproduction, have not yet been reported in other vertebrates and invertebrates. It is therefore still largely unclear how widely rGCs are utilized in the reproductive system of metazoans and when rGCs became involved in reproduction during the evolution of metazoans.

Basal metazoans include the nonbilaterian animal phyla Porifera (sponges), Cnidaria (corals, sea anemones, jellies, and hydras), Ctenophora (comb jelly), and Placozoa (*Trichoplax*). Because of their simplicity of body plan and phylogenetic position, these metazoans are regarded as evolutionarily primitive animals^29^. Studies on these animals are thought to be informative for speculation on the evolutionary traits of biological characteristics that exist in present-day metazoans^30^. Although the available genomic and transcriptomic databases of basal metazoans have been increasing^31–38^, no published studies are available for describing the characterization of rGCs in any basal metazoans.

The present study attempted to investigate the existence and expression of rGCs, particularly if rGCs are involved in the reproduction of a basal metazoan, phylum Cnidaria, using a gonochoric stony coral, *Euphyllia ancora. E. ancora* possesses relatively large polyps (3–5 cm in diameter) that allow us to isolate specific types of tissues (e.g., testis, ovary, or tentacle) from living polyps. Moreover, reliable molecular markers for germline cells have been identified, and their expression patterns in gametogenesis have been thoroughly characterized^39–41^. These attributes allow us to identify the tissue and cell types that have the expression of target gene products. We specifically explored the possible involvement of rGCs in the reproduction of the coral by cloning *rGC* genes followed by an investigation of the spatiotemporal expression of rGCs in coral polyp tissues.

## Results

### Identification of rGCs in *E. ancora* and phylogenetic analysis

*In silico* analysis of *E. ancora* transcriptome databases enabled us to identify 6 different sequences that contain the three conserved domains of rGCs: extracellular ligand binding domain (LBD), intracellular protein kinase-like homology domain (protein-KHD), and guanylyl cyclase (GC) domains (Supplementary Fig. S1). The sequences were then tentatively annotated as *GC-A*, *GC-B*, *GC-C*, *GC-D*, *GC-E* and *GC-F*, respectively (Supplementary Fig. S1). The similarities of LBD among these rGCs ranged from 18% to 42%, whereas those of the protein-KHD domain and GC domain were 41–72% and 57–86%, respectively (Supplementary Table S2). Phylogenetic analysis revealed that the 6 paralogous rGCs of *E. ancora*, supported by a strong bootstrap value (88~100%), were well-clustered with orthologous rGCs of other cnidarians (Supplementary Figs. S2 and S3). The phylogenetic analysis also showed that the cnidarian rGCs are not monophyletic and separated into two major groups, one of which contained GC-C, GC-D, GC-E and GC-G and was a sister group to protostome and echinoderm rGCs with 92% bootstrap value support. The other group contained cnidarian GC-A and GC-B and formed a specific cnidarian lineage, whereas GC-F was clustered together with placozoan rGCs (Supplementary Fig. S2). The homologous relationship between cnidarian rGCs and other taxa rGCs was not clear due to the low bootstrap values.

### Distribution of rGC transcripts in *E. ancora* polyp

**Figure 1:**
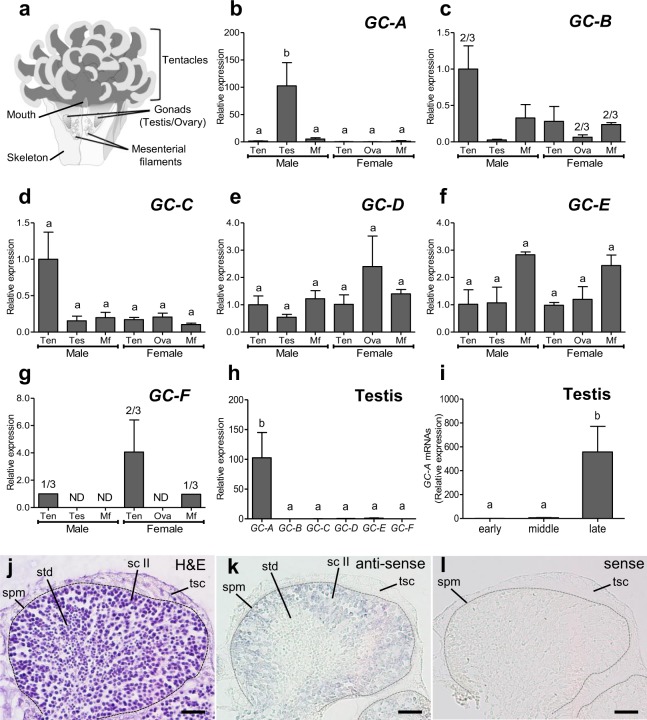
The tissue distribution of *rGC* transcripts in *E. ancora* polyp and localization of *GC-A-*expressing cells. (**a**) Schematic of the *E. ancora* polyp structure (modified from Shikina *et al*., ref.^57^). (**b**–**g**) Expression of *GC-A*, *GC-B*, *GC-C*, *GC-D*, *GC-E*, and *GC-F* mRNAs in the different parts of polyp tissues in male and female *E. ancora*. (**h**) Comparison of mRNA levels of the six *rGCs* in the testis of *E. ancora*. (**i**) Comparison of mRNA levels of *GC-A* in the testis containing different developmental stages of male germ cells. The samples investigated include tentacle (Ten), testis (Tes), mesenterial filament (Mf), and ovary (Ova). Data shown are the mean ± SE (n = 3 colonies) relative to the Ten group. Groups with different letters are significantly different (P < 0.05). Because of the low expression levels of *GC-B* (**c**) and *GC-F* (**j**) mRNA, the transcripts were only detected in 1 out of 3 colonies examined (1/3) or 2 out of 3 colonies examined (2/3). ND, not detected. Localization of *GC-A*-expressing cells in *E. ancora* testis (**j**–**l**). Sequential sections were stained with hematoxylin-eosin (**j**), hybridized to an antisense *GC-A* probe (**k**), or hybridized to a sense *GC-A* probe (**l**). A fine dotted line distinguishes the edge of spermary and testicular somatic tissue. The *GC-A* signal was detected mainly in secondary spermatocytes and faintly in spermatids. spm, spermary; tsc, testicular somatic cell; sc II, secondary spermatocyte; std, spermatid. Scale bar, 20 μm.

To explore the possible involvement of rGCs in the sexual reproduction of corals, different parts of polyp tissues (tentacle, mesenterial filament, and developing testis/ovary) were isolated from *E. ancora* polyps of both sexes (Fig. 1a), and the expression levels of 6 *rGC* genes in each tissue were investigated by qRT-PCR analysis. It was found that *GC-A* was almost specifically expressed in the testis (Fig. 1b). *GC-B*, *GC-C*, *GC-D*, and *GC-E* were not specifically expressed in the testis/ovary (Fig. 1c–f), and GC-F transcripts were not detected in the gonads and some other specimens (Fig. 1g). Comparison of the expression levels among 6 paralogous *rGCs* in the testis showed that the expression level of *GC-A* was notably higher compared to that of other *rGCs* (Fig. 1h). The qRT-PCR of cDNA from testes samples isolated at different developmental stages showed that the expression levels of *GC-A* significantly increased as male colonies approached the maturation period (Fig. 1i). An ISH with an antisense probe revealed that *GC-A* was expressed in male germ cells but not testicular somatic cells (Fig. 1k). The *GC-A* signal was detected mainly in the secondary spermatocytes and faintly in the spermatids. No signal was detected when the sense probe was applied (Fig. 1l).

### Elucidation of the cDNA sequence of GC-A and its sequence analysis

Based on the expression profile, the present study focused on GC-A and performed further investigation. The full-length sequence obtained by RACE-PCR was 4,791 bp in length and contained an open reading frame of 3,147 bp corresponding to 1,049 amino acid residues. Sequence analysis predicted the presence of a signal peptide at the N-terminal region, seven N-glycosylation sites, and 30 phosphorylation sites by serine, threonine, and/or tyrosine kinases (Supplementary Fig. S4). The predicted molecular mass was 117 kDa. Alignment of the catalytic GC domain of coral GC-A and other known active receptor GCs revealed classical guanylate cyclase sequence and ion (magnesium) binding site (Supplementary Fig. S5).

### Expression of GC-A protein in *E. ancora* male germ cells

**Figure 2:**
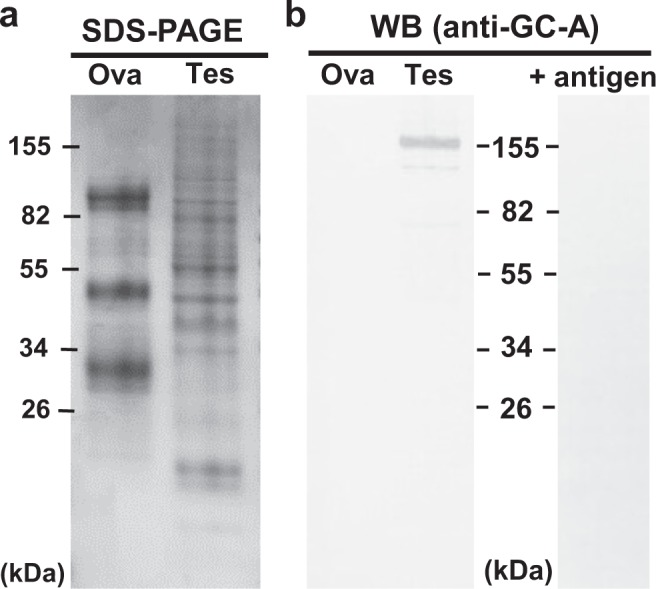
Evaluation of the anti-GC-A antibody specificity and GC-A expression in the developing gonads. The antibody was used for the analysis with or without preadsorption of its peptide antigen. (**a**) SDS-PAGE of protein extracts prepared from developing ovaries (Ova) and testes (Tes) of *E. ancora* that were collected in April (1 month before spawning). Markers with molecular sizes are shown. (**b**) Western blotting of the same protein extracts shown in (**a**), probed with an anti-GC-A antibody. An immunoreactive band at 155 kDa is shown. Note that the immunoreactivity was completely eliminated by the preadsorption of the antibody with the peptide antigens (+antigen). The amount of protein used was 10 µg for each tissue.

For further expression analysis, we performed immunodetection of GC-A with anti-*E. ancora* GC-A antibody. Western blot analysis of developing testis protein showed a major band of 155 kDa (Fig. 2; Supplementary Fig. S6). No immunoreactivity was detected in the proteins from developing immature ovaries (Fig. 2; Supplementary Fig. S6), tentacles, mesenterial filaments of both sexes. This finding was almost in agreement with the tissue distribution pattern of GC-A transcripts as assessed by qRT-PCR analysis (Fig. 1b). The immunoreactive band could be completely eliminated by preadsorbing the antibody with the peptide antigen (Fig. 2; Supplementary Fig. S6), indicating that the antibody possesses specificity against *E. ancora* GC-A.

**Figure 3:**
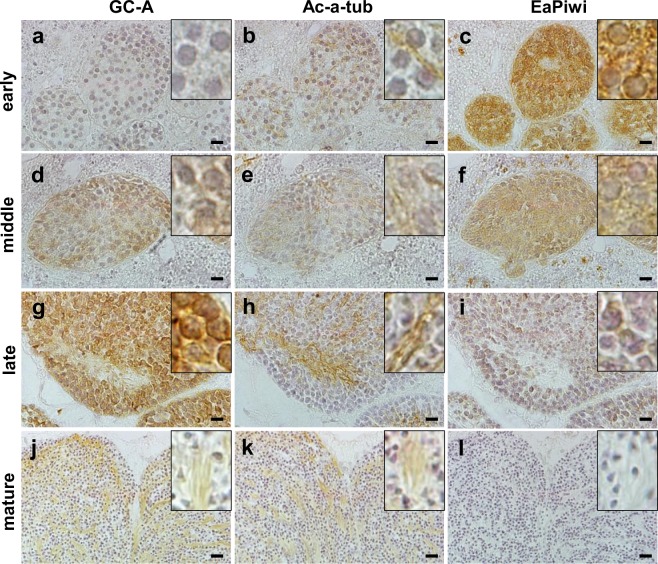
The expression of GC-A, Ac-α-Tu, and EaPiwi in male germ cells at different stages of spermatogenesis. (**a**,**d**,**g**,**j**) Expression profile of GC-A. (**b**,**e**,**h**,**k**) Expression profile of Ac-α-Tu. (**c**,**f**,**i**,**l**) Expression profile of EaPiwi. Sequential sections were used to analyse each stage of male germ cells. Scale bar, 20 μm.

**Figure 4:**
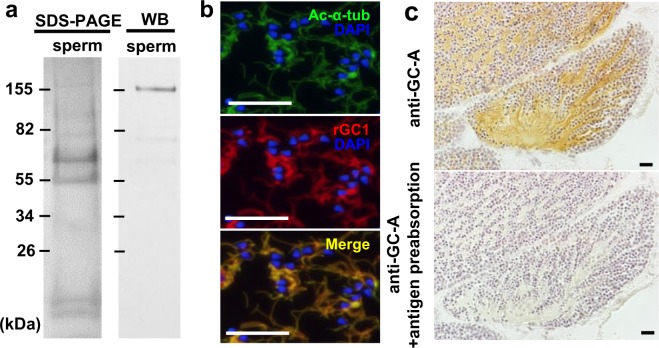
The localization of GC-A expression in the sperm. (**a**) SDS-PAGE and western blotting of sperm protein. Molecular size markers are shown. An immunoreactive band at 155 kDa is shown. (**b**) Double immunofluorescence detection of acetylated-alpha-tubulin, Ac-α-Tu (upper, green) and GC-A (middle, red) in the flagella of sperm in a paraffin section of *E. ancora* testis. Ac-α-Tu and GC-A were colocalized in the sperm flagella (lower, merge). (**c**) Immunohistochemical analysis of a mature testis. The immunoreactivity was concentrated in the sperm flagella and almost eliminated by the preadsorption of the antibody with the peptide antigens. The amount of protein used was 10 µg for each tissue. Scale bar, 20 μm.

Subsequently, the expression of GC-A protein in different developmental stages of male germ cells was investigated by immunohistochemical analysis. Ac-α-Tu and EaPiwi were used as markers for flagella on male germ cells and early-stage germ cells, respectively. The immunoreactivity of GC-A (ir-GC-A) was not detected in spermatogonia (early, Fig. 3a–c). The ir-GC-A began to be detected in the cytoplasm of primary spermatocytes (middle, Fig. 3d–f) and became stronger in the secondary spermatocytes and spermatids (late, Fig. 3g–i). The ir-GC-A was eventually localized to the sperm flagella but not to the sperm head (mature, Fig. 3j–l). Western blotting further demonstrated the presence of an immunoreactive band of GC-A in the protein extracted from released sperm (Fig. 4a; Supplementary Fig. S6). Colocalization of GC-A and Ac-α-Tu in sperm flagella was further confirmed by immunofluorescent analysis (Fig. 4b). The immunoreactive signal could be completely eliminated by preadsorbing the antibody with the peptide antigen (Fig. 4c).

### Expression of GC-A in the sperm/male germ cells of other corals

**Figure 5:**
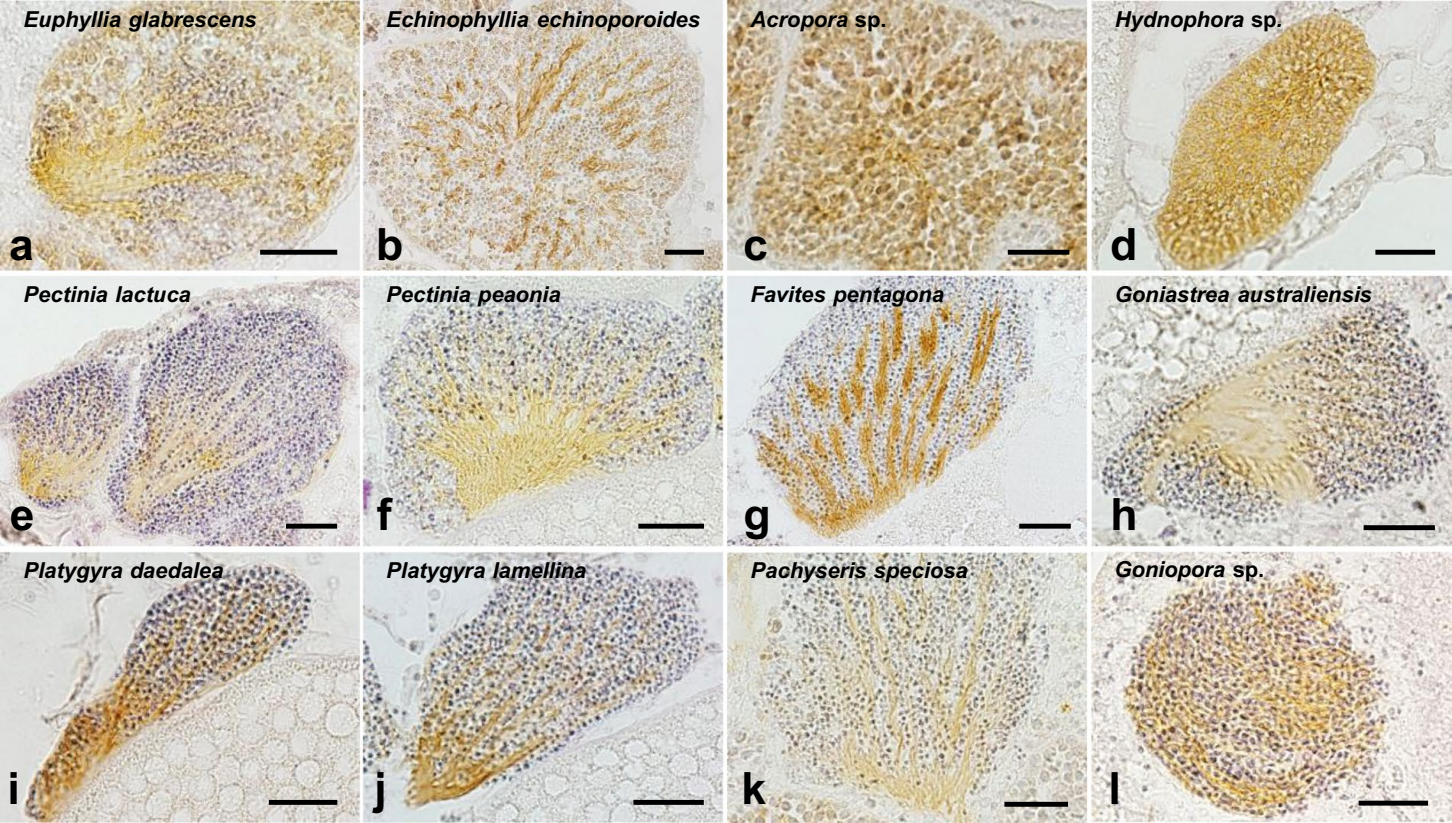
Presence of GC-A in the male germ cells of different stony corals as assessed by immunohistochemistry. (**a**) Sperm of *Euphyllia glabrescens*, (**b**) Spermatids of *Echinophyllia echinoporoides*, (**c**) Spermatogonia/primary spermatocytes of *Acropora* sp., (**d**) Spermatogonia/primary spermatocytes of *Hydnophora* sp., (**e**) Sperm of *Pectinia lectuca*, (**f**) Sperm of *Pectinia peaonia*, (**g**) Sperm of *Favites pentagona*, (**h**) Sperm of *Goniastrea australiensis*, (**i**) Sperm of *Platygyra daedalea*, (**j**) Sperm of *Platygyra lamellina*, (**k**) Sperm of *Pachyseris speciosa*, (**l**) Sperm of *Goniopora* sp. Scale bar, 20 μm.

Immunohistochemical analysis of other stony corals showed that the immunoreactivity was detected in the sperm flagella or male germ cells of a total of 12 species of stony corals, including the Euphyllidae family (Fig. 5a), the Lobophylliidae family (Fig. 5b), the Acroporidae family (Fig. 5c), the Merulinidae family (Fig. 5d–j), the Agariciidae family (Fig. 5k), and the Poritidae family (Fig. 5l).

## Discussion

By combining a transcriptome database search and expression analyses, we successfully demonstrated the presence of rGCs in the reproductive tissues/cells of an anthozoan cnidarian *E. ancora*. One of the identified rGCs, GC-A, is highly and almost exclusively expressed in the testis. Interestingly, GC-A was localized in the cytoplasm of spermatocytes and eventually congregated on the sperm flagella during spermiogenesis. These findings suggest that GC-A is involved in the regulation of sperm activity and/or functions (e.g., fertilization) in corals. To the best of our knowledge, this study is the first to perform molecular identification and characterization of rGCs in cnidarians and to provide evidence for the possible involvement of rGCs in the reproduction of basal metazoans.

Our sequence analysis of *E. ancora* GC-A showed the presence of signal peptide at the N-terminal region, transmembrane domain between LBD and intracellular protein-KHD, and catalytic GC domain. This suggested that identified *E. ancora* GC-A is a membrane-bound receptor GC. In addition, as shown in the known active receptor GCs in mammals and echinoderms^8,9,42^, *E. ancora* GC-A has been confirmed to contain conventional guanylate cyclase and consensus ion binding sites as well as residues important for catalytic activity and nucleotide binding. For the further characterization, the biological and/or physiological functions of *E. ancora* GC-A should be addressed by determination of GC activity and cGMP accumulation in future studies. Moreover, localization of GC-A on the membrane of secondary spermatocytes and sperm flagella of *E. ancora* also needs to be confirmed by immunoelectron microscopy.

The GC-A protein was expressed abundantly on the coral sperm flagella, which was consistent with the expression profile of echinoderm sperm flagella rGCs^8,9^. However, phylogenetic analysis showed that *E. ancora* GC-A appeared in the cnidarian-specific rGC lineage and was phylogenetically distinct from the sperm-associated rGCs in echinoderm and mammals. These findings imply two possible evolutionary scenarios for the involvement of rGCs in metazoan reproduction: (1) Sperm-associated rGCs originated from some common ancestor predating the divergence of the cnidarian and bilaterian lineages; however, they were lost in some invertebrate lineages, including model invertebrates, during the evolutionary processes^31,43,44^. (2) Sperm-associated rGCs have been independently acquired in some specific animal lineages, such as anthozoan cnidarians, echinoderms, and mammals. Further exploration of sperm-associated rGCs in all other major metazoan groups, including other basal metazoans, would provide further insights into the appearance and evolution of sperm-associated rGCs in metazoan reproduction.

The anti-*E. ancora* GC-A antibody was produced against a C-terminal region of GC-A, which is well-conserved among stony corals (79% to 86% sequence identity, see Supplementary Fig. S7), suggesting that the antibody can be used for the detection of GC-A in other stony corals. Indeed, our immunohistochemical analysis of GC-A in other corals with the antibody showed immunoreactivity in the sperm flagella or male germ cells, similar to the expression pattern of GC-A in *E. ancora*. This suggests that the antibody most likely showed reactivity to the GC-A of other corals, and that GC-A is expressed in the sperm flagella in a variety of stony corals. Besides, the antibody most likely does not show reactivity to other rGCs in *E. ancora* because of the low sequence identity (Supplementary Fig. S8). The sequence similarity of C-terminal epitope between GC-A and GC-B is relatively high (71%); however, no anti-*E. ancora* GC-A immunoreactivity was detected in the male tentacle where has the higher expression of *GC-B* transcripts (Supplementary Figs. S9 and S10).

The molecular mass of *E. ancora* GC-A protein assessed by WB analysis was 155 kDa, which was not in agreement with the predicted molecular mass of 117 kDa. This inconsistency may be due to the PTM of proteins, such as phosphorylation and glycosylation. Indeed, PTMs play important roles in cellular functions^45–47^ and commonly occur on cell surface receptors, including mammalian and echinoderm membrane-bound rGCs^3,46–52^. Our sequence analysis of *E. ancora* GC-A found the existence of a considerable number of putative PTM sites. A mass spectrometric analysis of purified *E. ancora* GC-A protein will reveal the presence of PTMs and clarify this inconsistency in future studies.

In cnidarians, although the presence of several types of SAAF has been demonstrated in hydrozoa^53^ and stony corals^54,55^, no receptor for SAAF has been identified thus far. The GC-A would be a promising candidate as a chemoreceptor for SAAF in corals. Future identification of SAAF of *E. ancora* by sperm chemotaxis assay, as well as demonstration of the binding between SAAF and GC-A, is necessary to reveal the function of GC-A in coral fertilization.

## Methods

### Sample collection

*E. ancora* colonies were labeled and collected by scuba divers at different times during 2016–2018 at Nanwan Bay, southern Taiwan (21°57′N, 120°46′E). *Euphyllia glabrescens* colonies were also collected at the same site. Collection of corals was permitted by the administration office of Kenting National Park (issue number: 1010006545). Another 11 coral species (*Echinophyllia echinoporoides*, *Acropora* sp., *Hydnophora* sp., *Pectinia lectuca*, *Pectinia peaonia*, *Favites pentagona*, *Goniastrea australiensis*, *Platygyra daedalea*, *Platygyra lamellina*, *Pachyseris speciosa*, and *Goniopora* sp.) were collected at Pitouchiao (25°13′N, 121°91′E) near northern Taiwan. These animals were selected because they were abundant at the sampling site. The collection was permitted by fisheries and fishing port affairs management office, New Taipei city government (issue number: 1063334179). Unfertilized eggs and sperm of *E. ancora* were obtained from colonies that were collected one week before the predicted spawning date and subsequently kept at an aquarium in National Taiwan Ocean University (NTOU). All of the rearing procedures were in accordance with the guidelines for the Institutional Animal Care and Use from NTOU.

### Cloning and sequencing of rGCs in *E. ancora*

Six putative *rGC* sequences were identified from the transcriptome databases of *E. ancora* polyps (Shikina and Chang, unpublished) by local BLAST search using human *GC-A, GC-B* and sea urchin resact receptor (GenBank accession numbers: NP_000897, NP_003986, ADM67560, respectively) sequences as queries. An NCBI conserved domain search was used to predict the conserved domains in the sequences. ClustalW was used to calculate the similarities among *E. ancora* rGC paralogs. To obtain the complete cDNA sequence of the *GC-A* gene, total RNA was extracted with TRIzol Reagent (Life Technologies, Carlsbad, CA) from the isolated testes that were collected in March. The SMART RACE cDNA amplification kit (Clontech, Mountain View, CA) was used for cDNA synthesis and 5′ and 3′ amplification of the cDNA ends. Specific sets of primers are shown in Supplementary Table S1. PCR products were electrophoresed in 1.5% agarose gel, purified, and sequenced with an ABI 3730xl DNA Analyzer (Life Technologies). Sequences were analysed with GENETYX version 11.0 (GENETYX, Tokyo, Japan). PROSITE (https://prosite.expasy.org/) was used for the prediction of posttranslational modification (PTM) sites. The molecular mass was predicted using ExPASy Compute pI/Mw (http://web.expasy.org/compute_pi/).

### Phylogenetic analysis

A subset of the GC member sequences from different animals was retrieved from GenBank (Supplementary Table S3) and phylogenetically compared with *E. ancora* GC-A, GC-B, GC-C, GC-D, GC-E, and GC-F (GenBank accession numbers MH894389, MH894390, MH894391, MH894392, MH894393, and MH894394, respectively). The sequences were aligned with MUSCLE, and the neighbor-joining phylogenetic trees were constructed by MEGA X using both the JTT matrix-based and p-distance models^56^.

### Expression profile of rGC transcripts

Tentacles, mesenterial filaments, immature testes containing spermatogonia and primary spermatocytes, and immature ovaries containing developing oocytes were isolated under a stereomicroscope from 6 different colonies (3 males and 3 females) collected in March. Testes containing early, middle, and late stages of male germ cells were collected in December, February, and April, respectively. All tissues were snap-frozen immediately after isolation by liquid nitrogen and preserved at −80 °C. Total RNA was extracted as described above, and the cDNA was synthesized using 2 μg of DNase-treated RNA with Superscript III reverse transcriptase (Invitrogen, Carlsbad, CA) according to the manufacturer’s instructions. The expression levels of *rGC* mRNA were determined by quantitative RT-PCR (qRT-PCR) according to the conditions described elsewhere^57^. *β-actin* was used as a reference gene (GenBank accession No. JQ968408). Specific sets of primers are shown in Supplementary Table S1. Data were analysed with Bio-Rad iQ5 Manager (Bio-Rad Laboratories, Hercules, CA) according to the 2^−ΔΔCt^ method^58^.

### *In situ* hybridization

RNA probes were synthesized by *in vitro* transcription using a 545-bp partial *GC-A* cDNA fragment (nucleotide 1,137–1,681 of *E. ancora GC-A*) labeled with digoxigenin (DIG)-labeled uridine triphosphate (Roche, Mannheim, Germany) and T7 polymerase (Promega, Madison, WI). *In situ* hybridization (ISH) was performed according to the methodology described in our previous study^59^.

### Antibody production

A polyclonal antibody against *E. ancora* GC-A was generated according to the procedure described in our previous study^59^. A synthetic polypeptide C + MTYWLKGRDGFDKP (amino-acid residues 1,020–1,033 of *E. ancora* GC-A) conjugated with bovine serum albumin (BSA) was used as the antigen. The antibody was purified by passing a total of 15 ml of antisera of the immunized guinea pig through an affinity column containing 3 mg of the antigenic peptide (YaoHong Biotechnology Inc., Taipei, Taiwan).

### Immunohistochemistry and immunofluorescence

Tissue fixation and immunohistochemistry were performed according to the methodologies described elsewhere^59^. Anti-*E. ancora* GC-A antibody, anti-*E. ancora* Piwi antibody (EaPiwi)^40^ and anti-acetylated alpha-tubulin antibody (Ac-α-Tu; Santa Cruz Biotechnology, Santa Cruz, CA)^41^ were diluted 1:10,000, 1:4,000 and 1:4,000, respectively, in phosphate buffered saline with 0.1% Tween-20 (PBT) containing 2% skim milk and used for primary antibody reactions. A biotinylated goat anti-guinea pig IgG (Vector Laboratories, Burlingame, CA) or anti-mouse IgG antibody (Anaspec, San Jose, CA) was diluted 1:4,000 in PBT with 2% skim milk and used for secondary antibody reactions. Immunoreactivity was visualized with 3,3′-diaminobenzidine (DAB; Sigma-Aldrich, Steinheim, Germany). For immunofluorescence, histological sections of mature testis were incubated in a solution containing both anti-*E. ancora* GC-A and anti-Ac-α-Tu antibodies. Fluorescence was achieved by coincubation of the sections with Alexa Fluor 488-conjugated goat anti-mouse IgG and Alexa Fluor 546-conjugated goat anti-guinea pig IgG (Molecular Probes, Eugene, both diluted 1: 100). Nuclei were stained with 4′,6-diamidino-2-phenylindole (DAPI, Sigma-Aldrich, St. Louis, MO). The sections were observed and photographed with a microscope (IX71SF1, Olympus, Tokyo, Japan). To confirm the antibody specificity, the antibody was preadsorbed with 5 μg/ml of the antigen and used in the primary antibody reactions. All other procedures were performed similarly to the immunohistochemical protocol.

### SDS-PAGE and western blotting

Proteins were extracted with TRIzol reagent (Life Technologies, Carlsbad, CA) following the manufacturer’s protocol. Western blotting (WB) was performed according to our previous method^59^. The anti-*E. ancora* GC-A antibody was diluted in tris-buffered saline with 0.1% Tween-20 (TBST) containing 1% skim milk (1: 20,000) and used for the primary antibody reaction. Confirmation of the antibody specificity was performed similarly to the immunohistochemistry above. The results of SDS-PAGE and WB were imaged using a Biospectrum AC Imaging System (UVP LLC, Upland, CA) and a digital camera (DSC-RX100M2, Sony Corp., Tokyo, Japan), respectively.

### Statistical analyses

The data are shown as the standard error of the mean (SEM). Statistical significance indicated by different lowercase letters was determined using one-way ANOVA followed by Bonferroni’s posttest. Statistical significance was accepted at *P* < 0.05. All data analyses were performed using GraphPad Prism (v.5.00; GraphPad Software, San Diego, CA, USA).

## Supplementary information

Supplementary information

## Data Availability

All data generated or analysed during this study are included in this published article and its Supplementary Information Files.
