# Supplementary material for: Discovery of a receptor guanylate cyclase expressed in the sperm flagella of stony corals
Source: Sci Rep. 2019 Oct 10;9:14652. doi: 10.1038/s41598-019-51224-7 (PMC6787079; doi:10.1038/s41598-019-51224-7)
Supplement: Supplementary file 1 — Supplementary information [file 41598_2019_51224_MOESM1_ESM.pdf]

Title: Discovery of a receptor guanylate cyclase expressed in the sperm flagella of stony corals

Authors: Yan Zhang[1], Yi-Ling Chiu[2, 3], Chieh-Jhen Chen[1], Yu-Ying Ho[4], Chuya Shinzato[5], Shinya Shikina[1, 6*], Ching-Fong Chang[1, 4*]

Affiliations: [1]Center of Excellence for the Oceans, National Taiwan Ocean University, Keelung 20224, Taiwan; [2]Doctoral Program in Marine Biotechnology, National Taiwan Ocean University, Keelung 20224, Taiwan; [3]Doctoral Program in Marine Biotechnology, Academia Sinica, Taipei 11529, Taiwan; [4]Department of Aquaculture, National Taiwan Ocean University, Keelung 20224, Taiwan; [5]Atmosphere and Ocean Research Institute, The University of Tokyo, Chiba 277-8564, Japan; [6]Institute of Marine Environment and Ecology, National Taiwan Ocean University, Keelung 20224, Taiwan.

[*]**Correspondence**: Ching-Fong Chang, Department of Aquaculture, National Taiwan Ocean University, No. 2, Pei-Ning Rd., Keelung 20224, Taiwan. E-mail: B0044@email.ntou.edu.tw

[*]**Correspondence**: Shinya Shikina, Institute of Marine Environment and Ecology, National Taiwan Ocean University, No. 2, Pei-Ning Rd., Keelung 20224, Taiwan. E-mail: shikina@mail.ntou.edu.tw

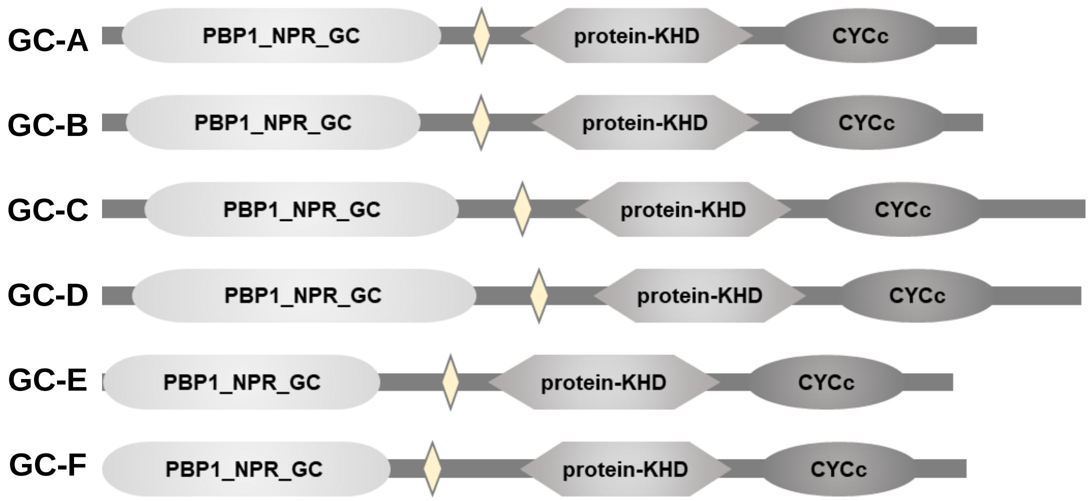

Supplementary Figure 1

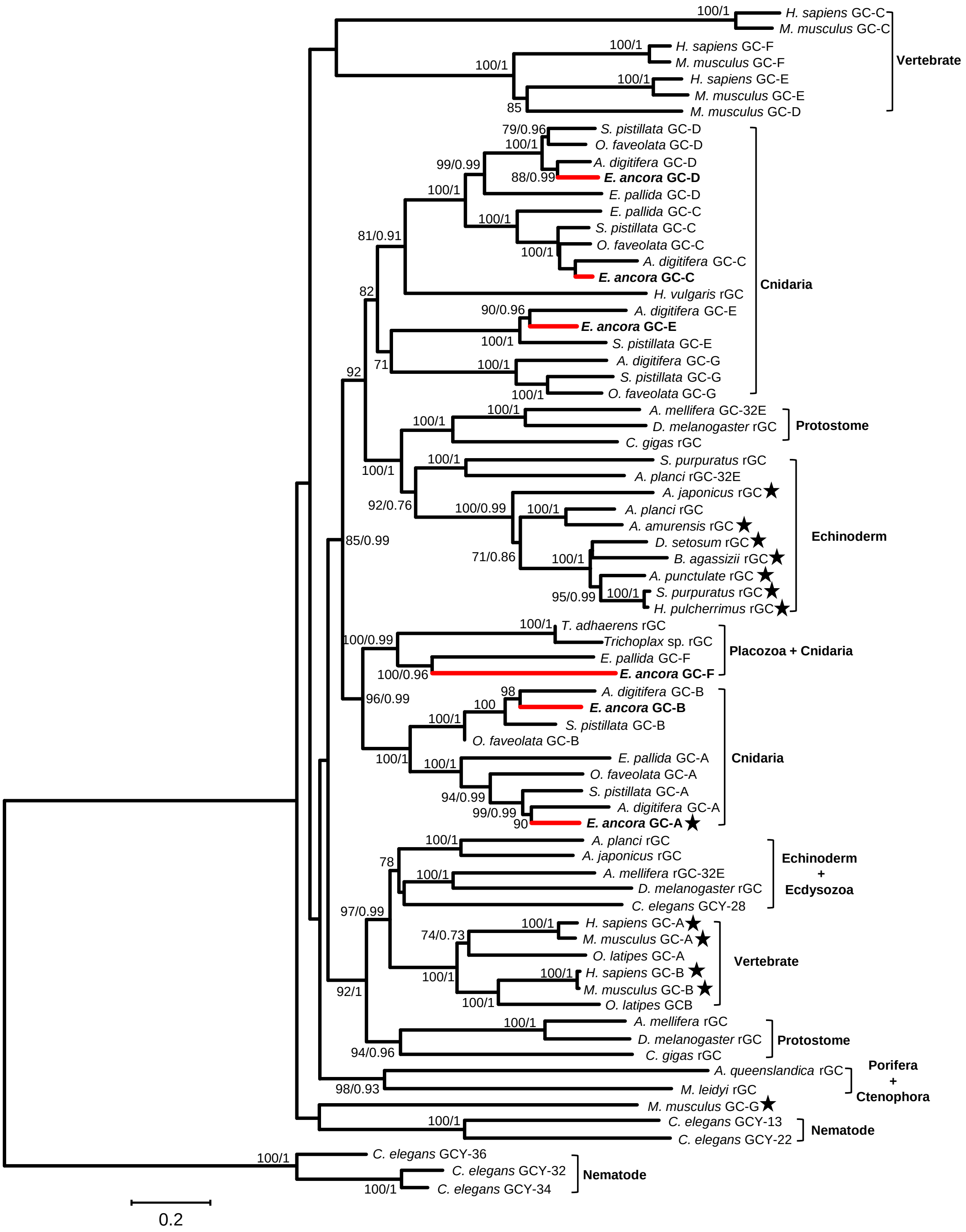

Supplementary Figure 2

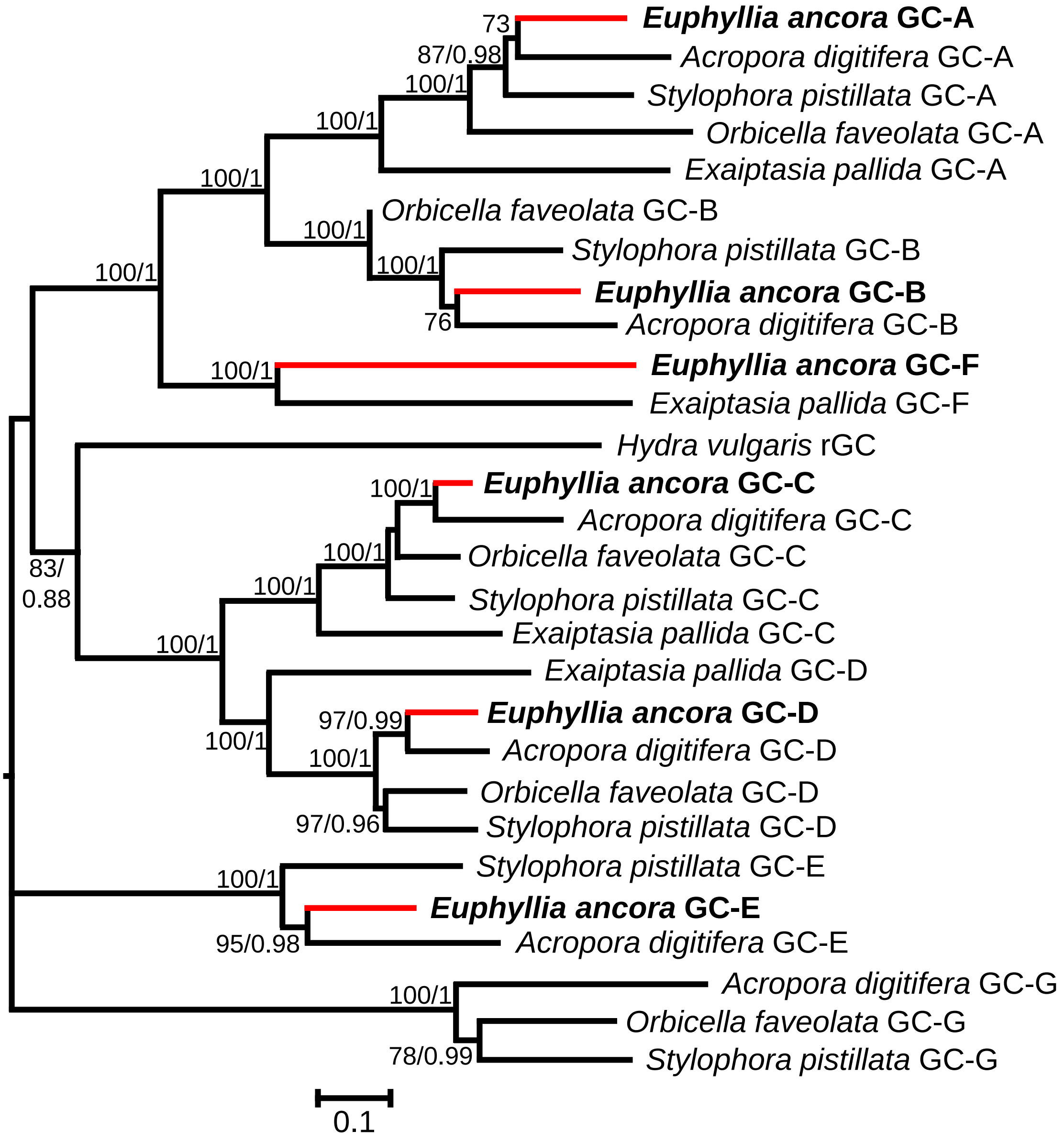

Supplementary Figure 3

MKIALFLWSI ICFVYLCHGR DIKMGIFVPF TGSWPGGPKM ASAILIAMDK VNQDPYWLQG 60

HTLTYLVRDG RCEAKSSLEI LADYYTVESP KVDVYIGPGC SAGCIPGAYL AAHWNIPMIS 120

WGCTASVLSD KTLYPYFVRT AGTYTGFGDL MRALLTQYSW DRAGIMASTE TVYSEIGNTV 180

KVSLEKDGKF SVPFFGSFDP GATDVSKLKS MLRSMATKAR VFLFLCYGGA FRKLMLSLYD 240

LGLLNGEYVV VTIETLPDSC KATASTQDGR DAEACKAYEG VIEVSQYIPD SQEYEDFTFA 300

VYN***RMPEMNY*** TMNAPNETNI FAAYLHDAIL LYAHAINESL NKNMSITDGK NISKSMIGKE 360

FLGVSGPVGI NEKGDRAASY RIQSFGSGMQ SKRVANFFGT TGQLQLLNKT IIWPGGTTKI 420

PIGRPACGFD NEFCKADAKE EDPIWPYILA GSLVVVLLVG VVVGVVLWQR KQAFEAALLA 480

RTWAVKYEDI KWPKNKGKLG SRKSMASMVG SERGSMDEIR GQIFTVLGTY EGNMVAVKNI 540

QKAKVNLDRD VLLELKEMKD LTHQNINTFV GACVDPGNIC ILTQYCNKGS LQDVLHNDTL 600

KLDWMFKMSI ASDIARGMNF LHNSPIQIHG NLKSSNVLID SRWTCKVTDH GLFLFKEGQE 660

IDVEAGSEAK YYDALWTAPE HILNNSFPRS QNGDVFSYGI ILSEILTRGL PYSMYEDFSA 720

RTIIDRVQKG VNPVLRPRIT KDLAEHHLFI QMMKQCWEQD ATLRPKFSEC LKLLKQMNKG 780

EDINIMDTMI TMMEKYTDHL EDIVAERTAE LAAEKAKTDE LLFRMLPRSV AEELKRGQPV 840

TAESFDSVTI FFSDIVGFTK IASDSTPLQV VDLLNDLYTC FDEIIDMHDV YKVETIGDSY 900

MVSSGLPTRN GNRHAGEIAN MSLDLLSAMT TFTVRHLPET QLQLRIGMHS GFVVAGVVGL 960

KMPRYCLFGD TVNYASRMES SGMALCIHVS PESKVILDQL GGYHLEERGE VEMKGKGKKM 1020

TYWLKGRDGF DKPLPDLSLQ AGMDEHTFK 1049

Supplementary Figure 4

```
H. sapiens GC-A       876 TIYFSDIVGFTALSAESTPMQVVTLLNDLYTCFDAVIDNFDVYKVETIGDAYMVVSGLPVRNGRLHACEV 945
M. musculus GC-A      872 ...............................................................Q...R.. 941
H. sapiens GC-B       861 ...................................I.......................G...QR..P.I 930
M. musculus GC-B      861 ...................................I.......................G...QR..P.I 930
M. musculus GC-G      901 ..F........K.CSL.S.L...K......SL..HT.QSH..............A....I...AQ..D.I 970
A. japonicus rGC      825 S.F............A...I...N.......L...I.A.Y.............L.....I...IF..GQI 894
A. amurensis rGC      873 ..F............A...I...N...A...T...T.S.Y.............L.....L...NR..GMI 942
D. setosum rGC        916 S.F............A...I...NM......L...I.A.Y.............L.....I...DN..GQI 985
B. agassizii rGC      927 S.F............A...I...N.......L...I.A.Y.............L.....I...DR..GQI 996
A. punctulate rGC     911 S.F............A...I...NM......L...I.A.Y.............L.....I...DR..GQI 980
S. purpuratus rGC     914 S.F............A...I...N.......L...I.S.Y.............L.....L...DR..GQI 983
H. pulcherrimus rGC   914 S.F............A...I...N.......L...I.S.Y.............L.....L...DR..GQI 983
E. ancora GC-A        849 ..F........KIASD...L...D..........EI..MH..........S...S....T...NR..G.I 918

H. sapiens GC-A       946 ARMALALLDAVRSFRIRHRPQEQLRLRIGIHTGPVCAGVVGLKMPRYCLFGDTVNTASRME 1106
M. musculus GC-A      942 ............................................................. 1102
H. sapiens GC-B       931 ...........S........HD.......V............................... 991
M. musculus GC-B      931 ...........S........HD.......V............................... 991
M. musculus GC-G      971 .T...H..SVTTH.Q.G.M.E.R.K....L.....V.....IT............M..... 1031
A. japonicus rGC      895 .ST.WH..ES.QT.IVP.KQE.K.K......S.SCV......T.................. 955
A. amurensis rGC      943 .SA.WH..EE.TT.VVP.KRD.K.K......S.SCV......T.................. 1003
D. setosum rGC        986 .ST.FH..ES.KN.IVP...DVF.K......S.SCV......T.............S.... 1046
B. agassizii rGC      997 .SA.WH..ES.K..VVP...DVF.K......S.SCV......T.............S.... 1057
A. punctulate rGC     981 .ST.YH..ES.KN.IVP...DVF.K......S.SCV......T.............S.... 1041
S. purpuratus rGC     984 .ST.HH..ES.KG.IVP.K.EVF.K......S.SCV......T.................. 1044
H. pulcherrimus rGC   984 .ST.HH..ES.KG.IVP.K.EVF.K......S.SCV......T.................. 1044
E. ancora GC-A        919 .N.S.D..S.MTT.TV..L.ET..Q....M.S.F.V..................Y..... 979
```

Guanylate cyclase

Supplementary Figure 5

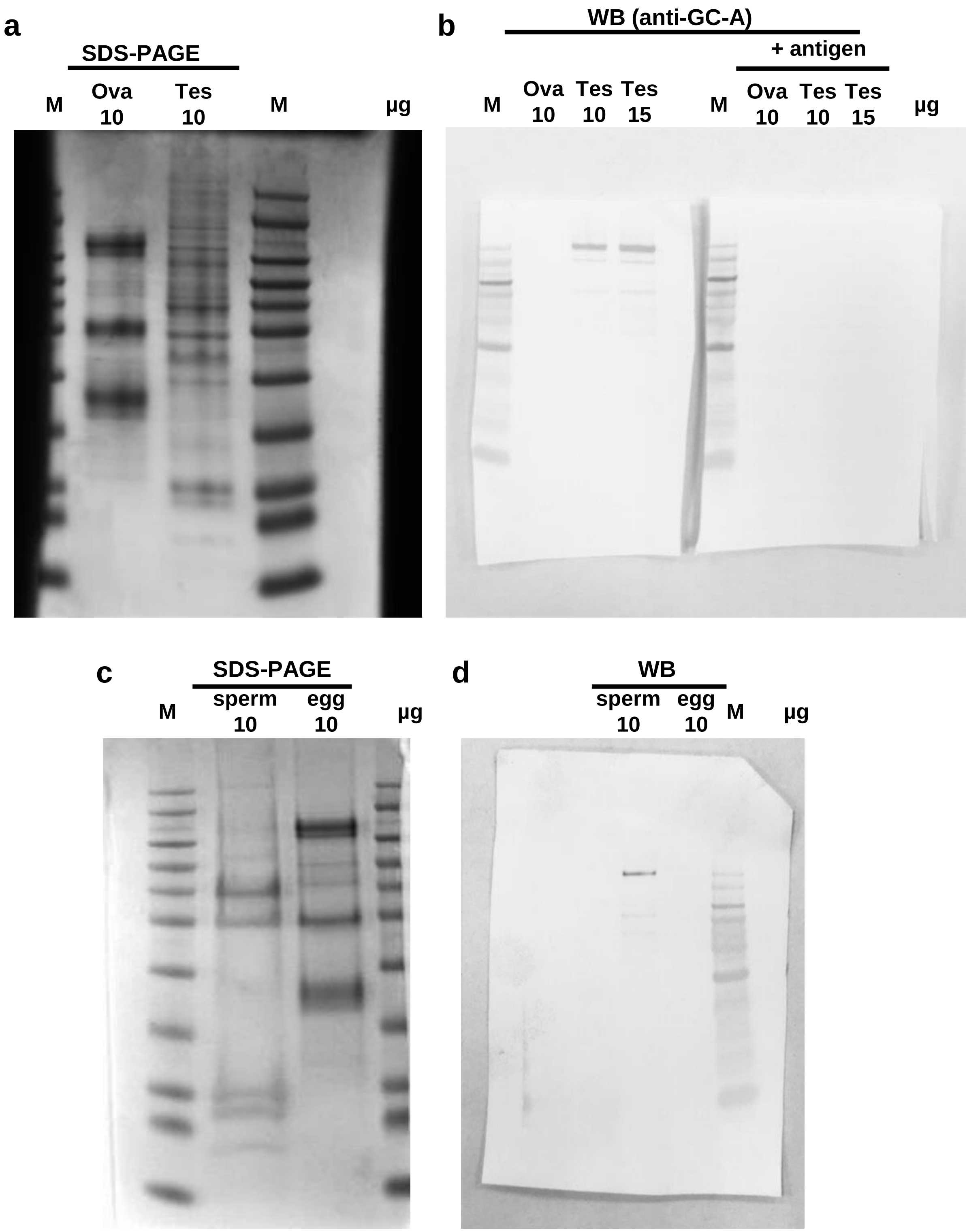

Supplementary Figure 6

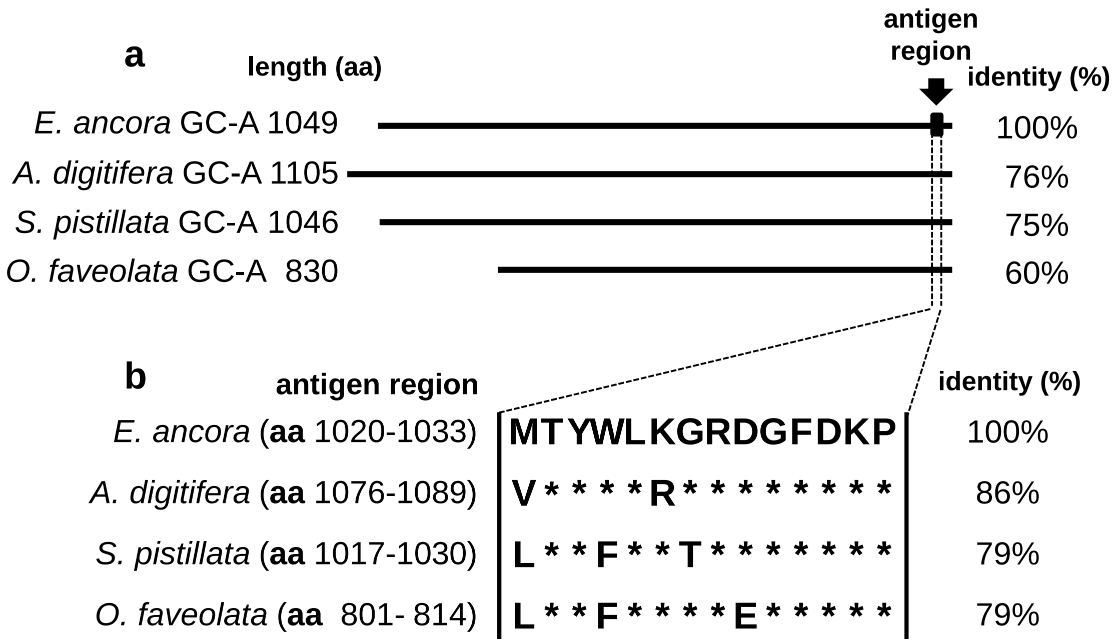

Supplementary Figure 7

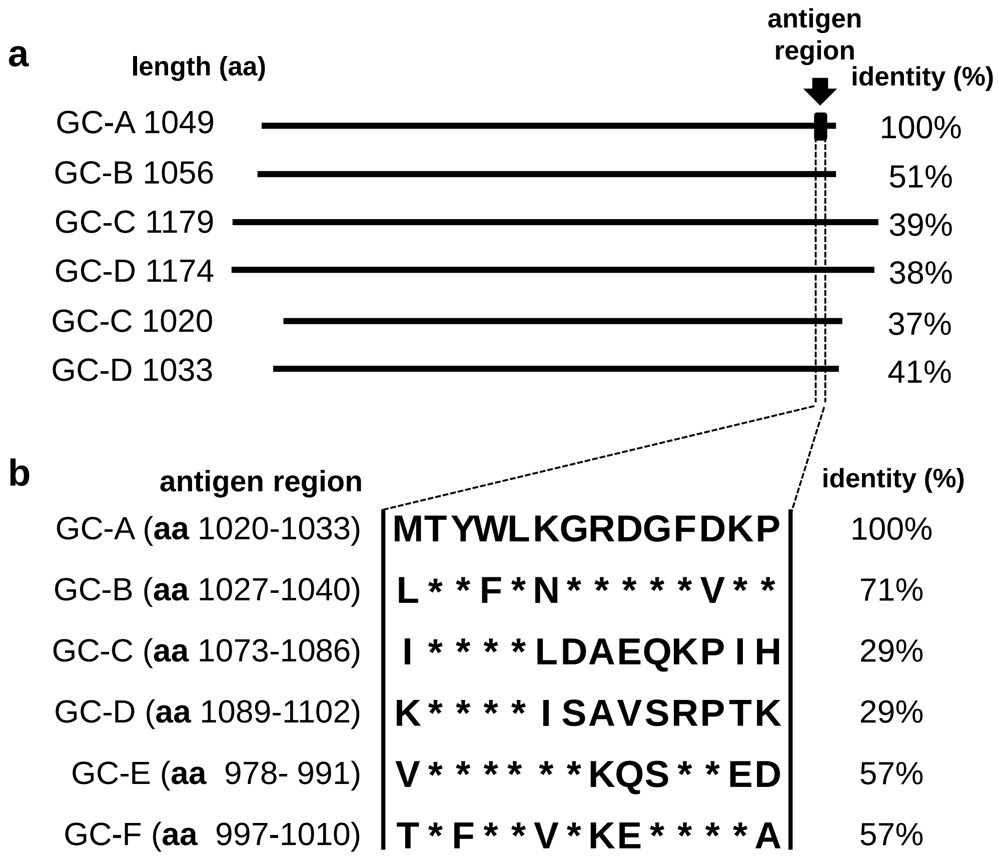

Supplementary Figure 8

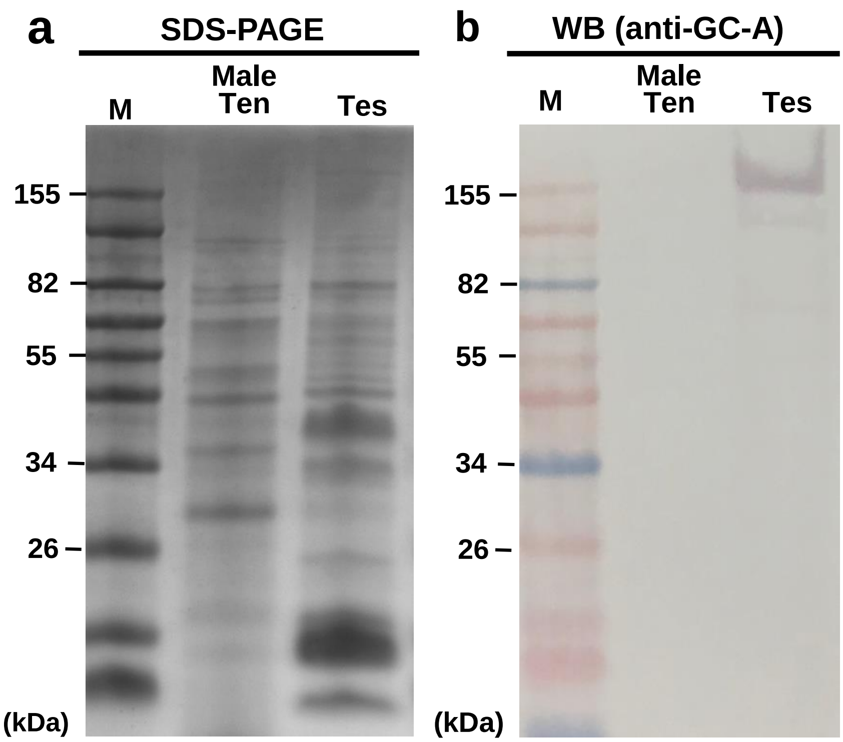

Supplementary Figure 9

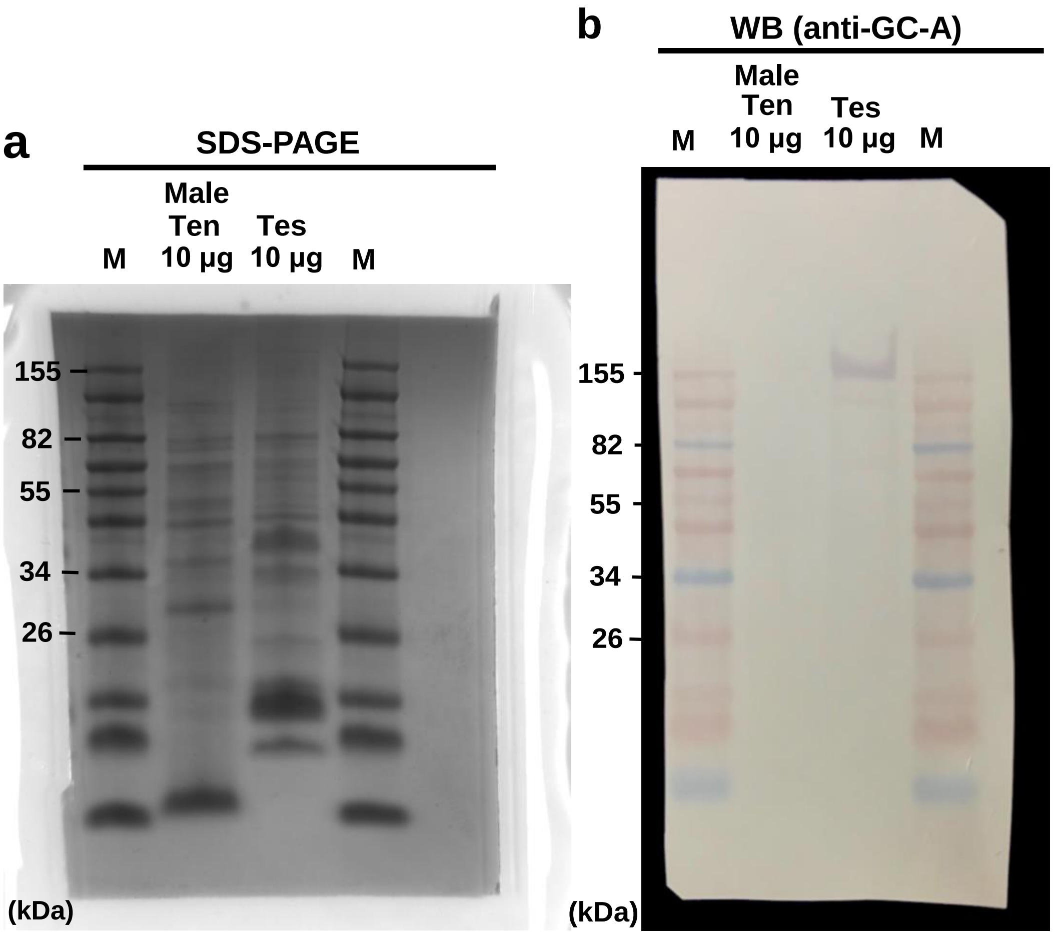

Supplementary Figure 10

Supplementary Figure 1.

Schematic figure depicting the domain structure of the identified rGC (GC-A, GC-B, GC-C, GC-D, GC-E, GC-F) proteins in *E. ancora*. The positions of the LBD, TM, protein-KHD, and GC domains are shown ordinally in the sequences.

Supplementary Figure 2.

A phylogenetic analysis of the deduced amino acid sequences of rGC-related proteins. A subset of rGC member sequences was retrieved from GenBank and aligned by ClustalW multiple sequence alignment with default parameters. The phylogenetic tree was constructed using the neighbor-joining method using the software MEGA X. Evolutionary distances were computed using the JTT matrix-based and p-distance model with the bootstrap test of 1000 replicates. The soluble guanylate cyclases GCY-32, GCY-34, and GCY-36 from *C. elegans* are used as outgroups. The number shown at each node represents the bootstrap value (JTT/p-distance); the branches shown correspond to values of 70% and higher. The testis- and/or sperm-localized rGCs based on experimental evidence are indicated by solid stars.

Supplementary Figure 3.

Phylogenetic analysis of the deduced amino acid sequences of rGC-related proteins from cnidarians. The phylogenetic tree was constructed using the neighbor-joining method using the software MEGA X. The number shown at each node represents the bootstrap value (%); only values of 70% and higher were shown.

Supplementary Figure 4.

Primary structure of the *E. ancora* GC-A. The N-terminal signal sequence (blue), the putative transmembrane segment (yellow shaded), the kinase homology (green), and the C-terminal catalytic domain (red) are indicated. Potential sites of ATP and nucleotide binding (red boxes), N-glycosylation (red stars), and predicted sites for phosphorylation by serine and threonine (asterisks) and tyrosine (underlined bold italic) kinases are indicated.

Supplementary Figure 5.

Multiple sequence alignment of the GC domains of *E. ancora* GC-A and known active receptor GCs. The species names are shown on the left. Blue boxes under inverted solid triangles indicate the characteristic ion binding site of GC. Core sequence of guanylate cyclase are shown within the red box. The identical amino acid to human GC-A is indicated by filled circles. GenBank accession numbers of the sequences used are as follows: *H. sapiens* GC-A NP_000897, *M. musculus* GC-A NP_032753, *H. sapiens* GC-B NP_003986, *M. musculus* GC-B NP_776149, *M. musculus* GC-G NP_001074545, *A. japonicus* rGC PIK35222, *A. amurensis* rGC BAB85468, *D. setosum* rGC BAA85332, *B. agassizii* rGC BAA75197, *A. punctulate* rGC ADM67560, *S. purpuratus* rGC NP_999705, *H. pulcherrimus* rGC BAA04660, and *E. ancora* MH894389.

Supplementary Figure 6.

Original pictures of SDS-PAGE and Western blotting used for Figure 2 and Figure 4. **a**) SDS-PAGE of protein extracts prepared from developing ovaries (Ova) and testes (Tes) of *E. ancora* that were collected in April (1 month before spawning). **b**) Western blotting of the same protein extracts shown in a), probed with an anti-GC-A antibody. **c**) SDS-PAGE of sperm and unfertilized egg protein. **d**) western blotting of same protein extracts shown in **c**). Numbers

represent the amount of protein used for each sample. M, molecular marker.

Supplementary Figure 7.

a) Schematic representation of GC-A sequences of stony corals. The species name and the length of each GC-A sequence are shown on the left. The amino acid identities of each GC-A calculated by pairwise comparison with *E. ancora* GC-A are shown on the right. The position of the antigen region of anti-*E. ancora* GC-A antibody (antigen region) and the corresponding position of each GC-A sequence are indicated with two vertical lines at the C-terminal region. **b**) Multiple sequence alignment of the antigen region. Amino acid identity is shown on the right relative to *E. ancora* GC-A. The identical amino acid is indicated by asterisks. aa, amino acid. GenBank accession numbers of the sequences are as follows: *E. ancora* MH894389, *A. digitifera* MK779950, *S. pistillata* XP_022796077, and *O. faveolata* XP_020632212.

Supplementary Figure 8.

a) Schematic representation of the identified 6 GC sequences in *E. ancora*. The length of each GC sequence is shown on the left. The amino acid identities of each GC calculated by pairwise comparison with GC-A are shown on the right. The position of the antigen region of anti-*E. ancora* GC-A antibody (antigen region) and the corresponding position of each GC sequence are indicated with two vertical lines at the C-terminal region. **b**) Multiple sequence alignment of the antigen region. The amino acid identity is shown on the right relative to GC-A. The identical amino acid is indicated by asterisks. aa, amino acid.

Supplementary Figure 9.

GC-A expression in the coral tentacles (Ten) and testes (Tes). **a**) SDS-PAGE of protein extracts prepared from tentacles and testis of *E. ancora* that were collected in May. Markers (M) with molecular sizes are shown. **b**) Western blotting of the same protein extracts shown in **a**), probed with an anti-GC-A antibody. The amount of protein used was 10 μg for each tissue.

Supplementary Figure 10.

Original pictures of SDS-PAGE and Western blotting used for Supplementary Figure 9. **a**) SDS-PAGE of protein extracts prepared from tentacles (Ten) and testes (Tes) of *E. ancora* that were collected in May. **b**) Western blotting of the same protein extracts shown in **a**), probed with an anti-GC-A antibody. Numbers represent the amount of protein used for each sample. M, molecular marker.

Title: Discovery of a receptor guanylate cyclase expressed in the sperm flagella of stony corals

Authors: Yan Zhang[1], Yi-Ling Chiu[2, 3], Chieh-Jhen Chen[1], Yu-Ying Ho[4], Chuya Shinzato[5], Shinya Shikina[1, 6*], Ching-Fong Chang[1, 4*]

Affiliations: [1]Center of Excellence for the Oceans, National Taiwan Ocean University, Keelung 20224, Taiwan; [2]Doctoral Program in Marine Biotechnology, National Taiwan Ocean University, Keelung 20224, Taiwan; [3]Doctoral Program in Marine Biotechnology, Academia Sinica, Taipei 11529, Taiwan; [4]Department of Aquaculture, National Taiwan Ocean University, Keelung 20224, Taiwan; [5]Atmosphere and Ocean Research Institute, The University of Tokyo, Chiba 277-8564, Japan; [6]Institute of Marine Environment and Ecology, National Taiwan Ocean University, Keelung 20224, Taiwan.

[*]**Correspondence**: Ching-Fong Chang, Department of Aquaculture, National Taiwan Ocean University, No. 2, Pei-Ning Rd., Keelung 20224, Taiwan. E-mail: B0044@email.ntou.edu.tw

[*]**Correspondence**: Shinya Shikina, Institute of Marine Environment and Ecology, National Taiwan Ocean University, No. 2, Pei-Ning Rd., Keelung 20224, Taiwan. E-mail: shikina@mail.ntou.edu.tw

Supplemental Materials

Table 1. Details of the primers used for *GC-A* cloning and expression analysis of *rGC*-family with the respective PCR conditions.

Purpose	Oligo name	Oligo sequence ('5 – '3)	Primer binding sites (amplicon size)	PCR conditions
GC-A UTRs amplification	npr1 3RACE-1st<br>npr1 3RACE-2nd<br><br>npr1 5RACE-1st<br>npr1 5RACE-2nd	GCC TAC AAG CCG GCA TGG ATG AAC ACA CT<br>CCA TGC TTG TCT TTG TAG CTC CCG ACA CT<br><br>AGC CAT GAT TCC TGC ACG ATC CCA AC<br>TGC CAT CCC TCA CCA AGT AGG TTA GCG TG	+3435 to +3463<br>+3713 to +3741<br><br>+798 to +823<br>+505 to +533	1x 94°C 5 min; 5x 94°C 30 sec, 70°C 30 sec and 72°C 150 sec; 5x 94°C 30 sec, 68°C 30 sec and 72°C 150 sec; 35x 94°C 30 sec, 66°C 30 sec and 72°C 150 sec; 1x 72°C 5 min
GC-A amplification (ORF)	npr1-230F<br>npr1-3755R	GCG ACA ATT TAC TGC TTA CC<br>CTG ACT TCG TAG TAA GTG TC	-93<br>+3433<br>(3526 bp)	1x 98°C 10 sec; 35x 98°C 10 sec, 55°C 15 sec, 72°C 2 min; 1x 72°C 3 min
GC-A qRT-PCR	GC-A-Fw<br>GC-A-Rv	GTG AGG GAT GGC AGA TGT GAA G<br>GTC CGA TAA CAC AGA GGC AGT G	(249 bp)	1x 95°C 3 min; 40x 95°C 10 sec, 60°C 30 sec

GC-B qRT-PCR	GC-B-Fw<br>GC-B-Rv	CTG ACC TCA GTA AAG CAG CGA G<br>CGG CCT AGC ATA TAG CGA ACT G	(105 bp)	1x 95°C 3 min; 40x 95°C 10 sec, 55°C 30 sec
GC-C qRT-PCR	GC-C-Fw<br>GC-C-Rv	CCA TGG AGA ACT TTG CAT CAA ACC<br>CTT CCA CAG TAG ACG TTT TAG CTC	(157 bp)	1x 95°C 3 min; 40x 95°C 10 sec, 60°C 30 sec
GC-D qRT-PCR	GC-D-Fw<br>GC-D-Rv	AAC CAG TGC CAG ATG AGG AGA C<br>AGA GTG TAG CCC GAG TTT TCC C	(138 bp)	1x 95°C 3 min; 40x 95°C 10 sec, 55°C 30 sec
GC-E qRT-PCR	GC-E-Fw<br>GC-E-Rv	TGG CCA CTA ATC CAG AGT TCG TAG<br>CCC CTC CAC CAA TAG TCA GGT TC	(130 bp)	1x 95°C 3 min; 40x 95°C 10 sec, 60°C 30 sec
GC-F qRT-PCR	GC-F-Fw<br>GC-F-Rv	GAC TAC AAT CAC TCC ACA GGC G<br>TGC CAG ATC AGT CTC ATA GCG A	(192 bp)	1x 95°C 3 min; 40x 95°C 10 sec, 55°C 30 sec
GC-A amplification (ISH probe)	ISH-Fw<br>ISH-Rv	CTG AAG CTT GCA AGG CCT ATG<br>CAG ACT CCC TGC CAA TAT GTA TG	(545 bp)	1x 94°C 5 min; 35x 94°C 30 sec, 56°C 30 sec, 72°C 45 sec; 1x 72°C 3 min

Table 2. Similarities of the full amino acid sequences and the three different domains between *E. ancora* rGCs.

%	GC-A	GC-B	GC-C	GC-D	GC-E	GC-F
**GC-A**	100					
**GC-B**	51 (42, 52, 76)	100				
**GC-C**	39 (25, 45, 67)	39 (24, 50, 66)	100			
**GC-D**	38 (18, 43, 69)	37 (20, 46, 67)	**56** (44, 72, 86)	100		
**GC-E**	37 (23, 41, 67)	38 (25, 41, 70)	44 (34,47,73)	41 (30, 49, 72)	100	
**GC-F**	41 (34, 45, 64)	40 (33, 48, 60)	38 (28, 48, 59)	37 (25, 47, 59)	**36** (27, 44, 57)	100

Sequence similarities among full-length sequences are values on the left; sequence similarities between LBD, PKc and GC domains are values within the right bracket. Highest similarities and lowest similarities of each domain are indicated by red, brown, and blue numbers, respectively.

Table 3. The names of the proteins analyzed in Supplementary Figure S2 and their GenBank accession numbers (ordinally from up to bottom).

	**Name**	**Accession No.**
1	*Homo sapiens* GC-C	NP_004954
2	*Mus musculus* GG-C	NP_001120790
3	*Homo sapiens* GC-F	NP_001513
4	*Mus musculus* GC-F	NP_001007577
5	*Homo sapiens* GC-E	NP_000171
6	*Mus musculus* GC-E	NP_032218
7	*Mus musculus* GC-D	NP_001124165
8	*Stylophora pistillata* GC-D	XP_022780263
9	*Orbicella faveolata* GC-D	XP_020613216
10	*Acropora digitifera* GC-D	XP_015762338
11	*Euphyllia ancora* GC-D	MH894392
12	*Exaiptasia pallida* GC-D	XP_020904271
13	*Exaiptasia pallida* GC-C	XP_020901343

14	*Stylophora pistillata* GC-C	XP_022780262
15	*Orbicella faveolata* GC-C	XP_020613215
16	*Acropora digitifera* GC-C	XP_015762337
17	*Euphyllia ancora* GC-C	MH894391
18	*Hydra vulgaris* rGC	XP_012560931
19	*Acropora digitifera* GC-E	XP_015779320
20	*Euphyllia ancora* GC-E	MH894393
21	*Stylophora pistillata* GC-E	XP_022780306
22	*Acropora digitifera* GC-G	XP_015779417
23	*Stylophora pistillata* GC-G	XP_022780225
24	*Orbicella faveolata* GC-G	XP_020613285
25	*Apis mellifera* GC-32E	XP_026295624
26	*Drosophila melanogaster* rGC	XP_001246742
27	*Crasspatrea gigas* rGC	XP_011450594
28	*Strongylocentrotus purpuratus* rGC	XP_782101
29	*Acanthaster planci* rGC-32E	XP_022091376
30	*Apostichopus japonicus* rGC	PIK35222

31	*Acanthaster planci* rGC	XP_022107162
32	*Asterias amurensis* rGC	BAB85468
33	*Diadema setosum* rGC	BAA85332
34	*Brissus agassizii* rGC	BAA75197
35	*Arbacia punctulate* rGC	ADM67560
36	*Strongylocentrotus purpuratus* rGC	NP_999705
37	*Hemicentrotus pulcherrimus* rGC	BAA04660
38	*Exaiptasia pallida* GC-F	XP_020898785
39	*Euphyllia ancora* GC-F	MH894394
40	*Trichoplax adhaerens* rGC	XP_002116283
41	*Trichoplax sp*. rGC	RDD47549
42	*Acropora digitifera* GC-B	XP_015754056
43	*Euphyllia ancora* GC-B	MH894390
44	*Stylophora pistillata* GC-B	XP_022790305
45	*Orbicella faveolata* GC-B	XP_020607147
46	*Exaiptasia pallida* GC-A	KXJ25520
47	*Orbicella faveolata* GC-A	XP_020632212

48	*Stylophora pistillata* GC-A	XP_022796077
49	*Acropora digitifera* GC-A	MK779950
50	*Euphyllia ancora* GC-A	MH894389
51	*Acanthaster planci* rGC	XP_022087153
52	*Apostichopus japonicus* rGC	BAA75224
53	*Apis mellifera* rGC	XP_026301092
54	*Drosophila melanogaster* rGC	NP_650505
55	*Caenorhabditis elegans* GCY-28	NP_001249628
56	*Homo sapiens* GC-A	NP_000897
57	*Mus musculus* GC-A	NP_032753
58	*Oryzias latipes* GC-A	NP_001098550
59	*Homo sapiens* GC-B	NP_003986
60	*Mus musculus* GC-B	NP_776149
61	*Oryzias latipes* GC-B	NP_001098128
62	*Apis mellifera* rGC	XP_001120461
63	*Drosophila melanogaster* rGC	NP_726013
64	*Crasspatrea gigas* rGC	XP_011440853

65	*Amphimedon queenslandica* rGC	XP_019849976
66	*Mneniopsis leidyi* rGC	AGB13751
67	*Mus musculus* GC-G	NP_001074545
68	*Caenorhabditis elegans* GCY-13	NP_506097
69	*Caenorhabditis elegans* GCY-22	NP_001263951
70	*Caenorhabditis elegans* GCY-36	NP_510557
71	*Caenorhabditis elegans* GCY-32	NP_506452
72	*Caenorhabditis elegans* GCY-34	NP_506319
